# Real-Time Callus Instance Segmentation in Plant Tissue Culture Using Successive Generations of YOLO Architectures

**DOI:** 10.3390/plants15010047

**Published:** 2025-12-23

**Authors:** Yunus Egi, Tülay Oter, Mortaza Hajyzadeh, Muammer Catak

**Affiliations:** 1 Department of Electrical and Electronics Engineering, Sirnak University, Sirnak 73000, Türkiye; 2 Department of Field Crops, Faculty of Agriculture, Sirnak University, Sirnak 73000, Türkiye; tulayoter.632@gmail.com (T.O.); m.hajyzadeh@gmail.com (M.H.); 3 College of Engineering and Technology, American University of the Middle East, Egaila 54200, Kuwait; muammer.catak@aum.edu.kw

**Keywords:** YOLO, instance segmentation, callus, plant tissue culture, deep learning, real-time inference, *Lens culinaris*

## Abstract

Callus induction is a complex procedure in plant organ, cell, and tissue culture that underpins processes such as metabolite production, regeneration, and genetic transformation. It is important to monitor callus formation alongside subjective evaluations, which require labor-intensive care. In this research, the first curated lentil (*Lens culinaris*) callus dataset for instance segmentation was experimentally generated using three genotypes as one data set: Firat-87, Cagil, and Tigris. Leaf explants were cultured on MS medium fortified with different concentrations of gross regulators of BA and NAA to induce callus formation. Three biologically relevant stages, the leaf stage, the green callus, and the necrosis callus, were produced. During this process, 122 high-resolution images were obtained, resulting in 1185 total annotations across them. The dataset was evaluated across four successive generations (v5/7/8/11) of YOLO deep learning models under identical conditions using mAP, Dice coefficient, Precision, Recall, and IoU, together with efficiency metrics including parameter counts, FLOPs, and inference speed. The results show that anchor-based variants (YOLOv5/7) relied on predefined priors and showed limited boundary precision, whereas anchor-free designs (YOLOv8/11) used decoupled heads and direct center/boundary regression that provided clear advantages for callus structures. YOLOv8 reached the highest instance segmentation precision with mAP50@0.855, while it matched the accuracy with greater efficiency and achieved real-time inference with 166 FPS.

## 1. Introduction

Plant tissue culture is an essential component of contemporary agricultural biotechnology, which makes it possible to achieve micro propagation, genetic transformation, haploid production, and the synthesis of valuable metabolites [1,2]. Among these methods, callus induction serves as the initial step toward somatic embryogenesis and plant regeneration. A callus is described as a cluster of undifferentiated plant cells under particular hormonal and nutritional environments [3,4]. Its ability to grow into a whole plant makes it essential for both fundamental plant science and practical biotechnology.

The lentil (*Lens culinaris*) is a vital global pulse crop prized for its high protein content and adaptability. However, it is especially difficult to work with in tissue culture, showing low regeneration rates and a strong dependence on the plant’s specific genetic makeup [5,6]. This makes the development of reliable callus induction methods critical for improving breeding and transformation. Manual visual inspection of a callus’s size, shape, and color is currently the standard for monitoring its formation. However, this method is subjective and unreliable, making it unsuitable for large-scale studies. To improve accuracy and efficiency in this research, we urgently need to develop automated, quantitative, and scalable evaluation tools. Another critical aspect is dataset availability. Unlike common benchmarks such as COCO or Pascal VOC [7], agricultural imaging datasets remain scarce, often limited to disease detection (e.g., PlantVillage) or UAV-based canopy monitoring.

Deep learning has revolutionized agricultural imaging, with Convolutional Neural Networks (CNNs) and one-stage detectors used for everything from disease identification to fruit counting and phenotyping [8]. The YOLO (You Only Look Once) family of models is particularly effective, which provides a strong balance of real-time speed and high accuracy. Later versions have introduced advanced features such as Cross-Stage Partial Darknet (CSPDarknet) backbones, Path Aggregation Network (PANet) style necks, and anchor-free heads, which utilize decoupled prediction together with Cross-Stage Partial blocks that use two Convolutions and Feature reuse (C2f) modules. The most modern designs, including EfficientRep and Parallel Spatial Attention, effectively balance precise segmentation with high-speed performance [9,10].

A significant development is the move from anchor-based heads (like those in YOLOv5/7) to anchor-free and decoupled designs (YOLOv8/11). Anchor-based models require predefined priors, whereas anchor-free variants predict centers and boundaries directly, which improves their ability to generalize. This distinction is especially relevant for irregular tissues such as callus, which present variable shapes that remain challenging for anchor-based models. For this reason, anchor-free segmentation frameworks show the most promise for biological imaging [11]. Despite focusing on object detection, instance segmentation is gaining importance because it provides pixel-level outlines rather than simple bounding boxes. This is especially useful for callus, which has irregular and often overlapping shapes. Agricultural research has already demonstrated the effectiveness of YOLO-based instance segmentation for tasks such as weed–crop differentiation and the assessment of lodged plant areas [12]. This proves that the technology is practical even in challenging field environments. These advances suggest that YOLO instance segmentation frameworks are a great fit for tissue culture analysis.

Building on this, the recent literature has further expanded YOLO’s role in agricultural and biological segmentation. Zhao et al. demonstrated that integrating the Segment Anything Model (SAM) with YOLOv8 could automatically segment leaf structures efficiently, which requires minimal manual annotation for real-time processing [13]. In the context of crop–weed differentiation, Zhang et al. presented F-YOLOv8n-seg, a compact segmentation model that reduced computational operations (FLOPs) by 26.7% while maintaining robust localization of weed meristems [14]. Similarly, Sonawane and Patil adapted YOLOv8 for crop–weed segmentation, proving its resilience to the variable illumination and background conditions found in field environments [15].

Horticultural applications have also pointed out mask quality. Chen et al. compared YOLOv8/11 for orchard fruitlet instance segmentation, which revealed that YOLOv11 improved mask precision at a decent computational cost [16]. Building on this direction, Ma et al. introduced YOLOv11-GSF for strawberry ripeness assessment, while Khan et al. developed a YOLOv11 framework for leaf segmentation across multiple crops with consistent performance across lighting conditions [17,18]. Segmentation was also applied to practical agricultural challenges such as disease and stress detection. Zhu et al. [19] created YOLOv8-CMS to identify citrus leaf diseases that led to Precision rates above 98% when grading symptomatic leaves. At the same time, Daraghmi et al. [20] demonstrated that combining UAV imagery with YOLOv8 makes it possible to pinpoint crop stress areas in real time, offering farmers a powerful tool for large-scale field assessment.

Anchor-free segmentation has gained significant attention in agricultural case studies, which has become an important research direction in general computer vision. Representative models such as FCOS [21], CenterNet [22], and DETR [23] remove the use of predefined anchor boxes. Instead, they predict object centers and masks directly, creating a simpler detection process. This approach laid the groundwork for the anchor-free strategies later used in YOLOv8/11. Their success in general-purpose segmentation underscores the relevance of anchor-free strategies for irregular biological objects such as callus tissues. Deep learning has likewise advanced biological and medical segmentation, where amorphous and heterogeneous structures resemble callus morphology. Caicedo et al. [24] evaluated several deep models for nucleus segmentation that produce valuable benchmarks. In parallel, Moen et al. [25] surveyed applications in microscopy and histology, which points out that Convolutional Neural Networks (CNN) consistently deliver higher accuracy and reproducibility than traditional approaches. Together, these works demonstrate a key point: frameworks that were first designed to segment natural images can be successfully adapted and extended to address the structural complexity of cellular and tissue samples.

Taken together, these studies confirm that anchor-free YOLO variants are increasingly applied to instance segmentation in agriculture, spanning weed control, leaf morphology, fruit detection, and disease monitoring, yet none address the segmentation of callus tissue in plant in vitro culture. The present work fills this gap by providing a curated callus segmentation dataset and a systematic benchmark across successive YOLO generations.

## 2. Results

### 2.1. Runtime, Convergence, and Training Dynamics

Evaluating modern YOLO architectures requires attention to both runtime efficiency and training behavior. This is especially important for applications that rely on real-time inference and consistent optimization within restricted computational settings, such as plant tissue culture monitoring. While runtime performance determines the practical feasibility of deployment, the trajectory of training curves provides insight into convergence speed, stability, and generalization capacity. To this end, we report measured training times and analyze the progression of mAP together with the major loss components across successive YOLO generations. On the NVIDIA Tesla T4 GPU (16 GB VRAM), the measured wall-clock times for 100 training epochs were YOLOv5—0.214 h (≈12.8 min); YOLOv7—0.652 h (≈39.1 min); YOLOv8—0.214 h (≈12.8 min); and YOLOv11—0.214 h (≈12.8 min). These results confirm that YOLOv7 is the slowest model due to its heavier architecture, which includes 37.9 M parameters and 141.9 G FLOPs. By contrast, the anchor-free designs of YOLOv8/11 sustain high efficiency without introducing runtime penalties.

**Figure 1:**
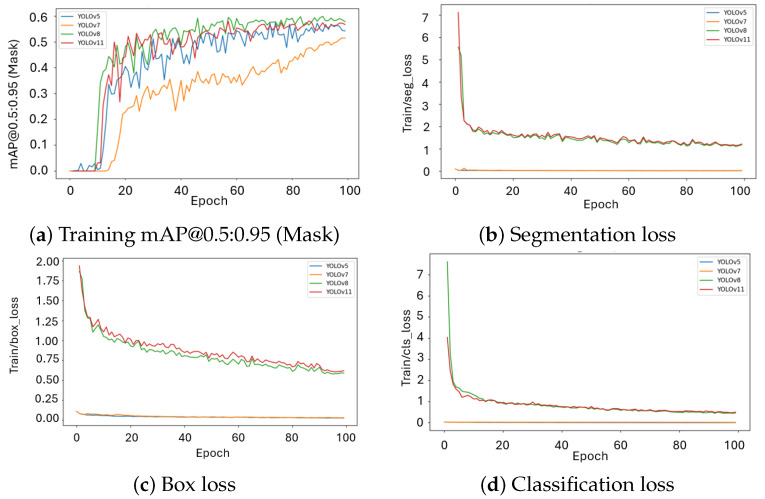
Training dynamics of YOLO models: mAP progression and core loss components.

The training curves (Figure 1a) show that YOLOv5/7 reach a plateau only after about 90 epochs. The gradual exponential decline with noticeable fluctuation points to a slower optimization with fewer stable gradient updates. Such tendencies are characteristic of anchor-based frameworks, where the bounding-box allocation procedure increases variance and hinders convergence.

In contrast, YOLOv8/11 reaches stability at approximately epoch 60. These outcomes reflect more efficient learning dynamics and indicate a stronger inductive bias, which facilitates rapid adaptation to intricate callus boundaries.

The segmentation loss (Figure 1b) drops quickly for YOLOv8/11, moving from high initial values to a stable range near 1.0–1.2. This pattern indicates effective separation of features and better refinement of segmentation masks. In contrast, YOLOv5/7 remain near zero, which is consistent with the limited design of their segmentation heads. For box regression loss (Figure 1c), YOLOv8/11 begin with higher values but gradually decline to around 0.75. In contrast, YOLOv5/7 remain very small across training, a result of both different loss-scaling conventions and the limited representational capacity of anchor-based localization. The classification loss (Figure 1d) further illustrates the difference between the two groups: YOLOv8/11 start at elevated levels (7–7.5) but progressively drop below 1.0, indicating stronger class separation and more effective optimization of category boundaries. By comparison, YOLOv5/7 stay near zero, suggesting that their simpler anchor-based classification head provides little contribution to discriminative learning. Overall, these results show that anchor-free models not only converge faster but also provide stronger optimization signals across segmentation, regression, and classification tasks.

In contrast, YOLOv8 reaches stability at approximately epoch 70, while YOLOv11 converges earlier, around epoch 60. These outcomes reflect more efficient learning dynamics and indicate a stronger inductive bias, which facilitates rapid adaptation to intricate callus boundaries. The segmentation loss (Figure 1b) drops quickly for YOLOv8/11, moving from high initial values to a stable range near 1.0–1.2. This pattern indicates effective separation of features and better refinement of segmentation masks. In contrast, YOLOv5/7 remain near zero, which is consistent with the limited design of their segmentation heads. For box regression loss (Figure 1c), YOLOv8/11 begins with higher values but gradually declines to around 0.6. In contrast, YOLOv5/7 remain very small across training, a result of both different loss-scaling conventions and the limited representational capacity of anchor-based localization. The classification loss (Figure 1d) further illustrates the difference between the two groups: YOLOv8/11 start at elevated levels (7–9) but gradually drop below 1.0, indicating stronger class separation and more effective optimization of category boundaries. By comparison, YOLOv5/7 stay near zero, suggesting that their simpler anchor-based classification head provides little contribution to discriminative learning. Overall, these results show that anchor-free models not only converge faster but also provide stronger optimization across segmentation, regression, and classification tasks.

**Figure 2:**
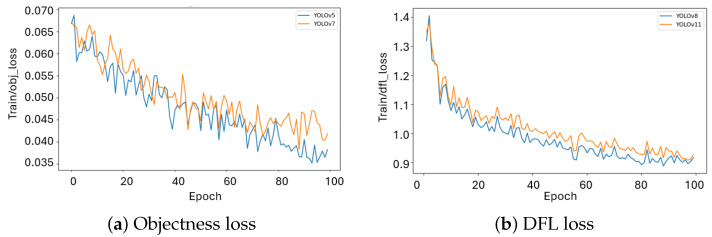
Training dynamics of YOLO models: auxiliary loss components (objectness and DFL).

Objectness loss (Figure 2a) appears only in YOLOv5/7, decreasing from about 0.69 to 0.4 but with noticeable noise across epochs. This instability reflects inherent drawbacks of anchor-based objectness scoring, in which predefined anchor grids frequently generate redundant or conflicting assignments, thereby weakening optimization stability. The noisy trend indicates that object presence is reinforced inconsistently, which results in weaker convergence and diminished robustness in complex scenarios.

In contrast, Distribution Focal Loss (Figure 2b), which is unique to YOLOv8/11, decreases smoothly from above 1.4 to below 1.0, showing the ability of anchor-free models to refine bounding-box distributions more reliably. Among the two, YOLOv8 exhibits a lesser dfl loss than YOLOv11, suggesting that its optimization pathway is less prone to variance and that its architecture enforces a more reliable alignment between predicted distributions and ground truth.

In summary, anchor-free YOLOv8/11 converge faster, maintain smoother loss behavior, and deliver stronger optimization than anchor-based YOLOv5/7, making them better suited for reliable real-time segmentation in plant tissue culture.

### 2.2. Segmentation Accuracy and Efficiency Metrics

Table 1 summarizes segmentation accuracy (mask mAP), efficiency metrics (parameter count, FLOPs, FPS), and training time. Unless otherwise specified, mAP refers to instance segmentation masks (denoted as (M) in logs).

Across all models, a clear divide emerged between anchor-based and anchor-free architectures. YOLOv5/7 was validated at mAP@0.5:0.95 values of 0.573 and 0.515, respectively. Although YOLOv7 improved relative to YOLOv5, its higher parameter count (37.9 M) and FLOPs (141.9G) did not translate into proportionate accuracy gains. This concludes that simply scaling up anchor-based networks is not an effective strategy for small, heterogeneous biological datasets.

**Figure 3:**
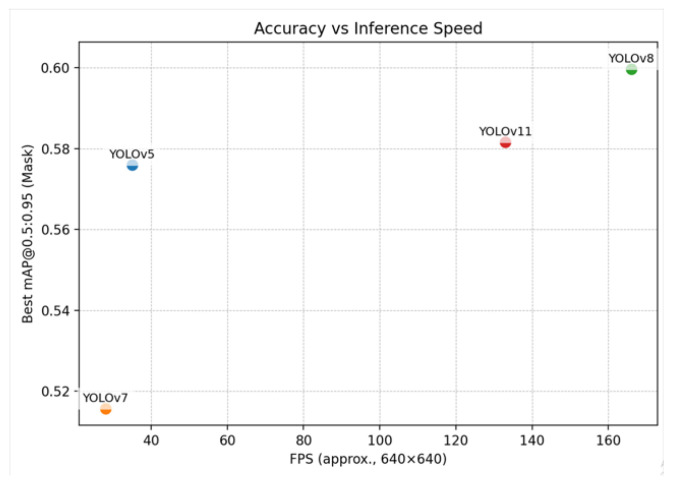
Accuracy vs. inference speed for YOLO variants on the lentil callus dataset. Best mask mAP@0.5:0.95 vs. FPS at 640×640 on an NVIDIA T4 (FP16, batch size 1, NMS included).

In contrast, anchor-free models delivered substantial gains. YOLOv8 achieved the highest overall validation accuracy (mAP@0.5:0.95 = 0.599), with YOLOv11 close behind at 0.581. YOLOv8 also demonstrated superior inference efficiency, reaching 166 FPS compared to 133 FPS for YOLOv11 (Table 1). This advantage stems from architectural refinements such as the EfficientRep backbone and Parallel Spatial Attention. The accuracy–speed trade-off (Figure 3) highlights that YOLOv8 provides the best overall balance for real-time laboratory deployment, while YOLOv11 remains a strong, closely performing alternative.

### 2.3. Class-Level Performance

**Figure 4:**
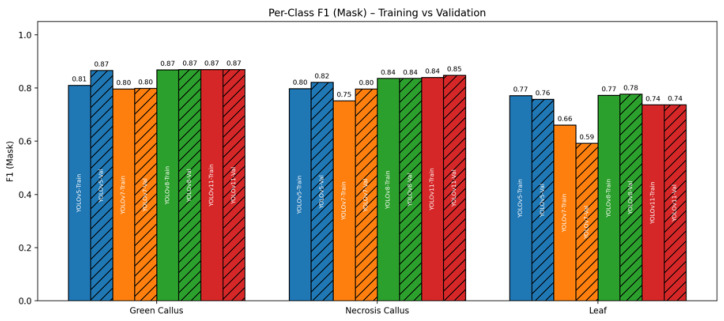
Combined per-class F1 (Mask) across YOLO variants. For each class (Green Callus, Necrosis Callus, Leaf), bars are grouped as YOLOv5/7/8/11 with Training (solid) and Validation (hatched) bars. Anchor-free models (YOLOv8/YOLOv11) achieve the best validation F1 on Green and Leaf, while all models perform highly on Necrosis. Labels inside bars indicate the model and split (e.g., YOLOv8-Val).

Figure 4 summarizes per-class mask F1 for green callus, necrosis callus, and leaf across all YOLO variants, showing both training (solid fill) and validation (hatched) results in a single chart. Three consistent patterns emerge:

(i) Green callus is the easiest class: All models reach F1 ≈ 0.81–0.87 on green callus, with the anchor-free models (YOLOv8/11) converging at 0.87 on both train and validation. This reflects the strong visual contrast of green tissue against the background.

(ii) Necrosis callus is moderately difficult: On the necrosis callus class, YOLOv11 attains the highest validation F1 (∼0.85) with YOLOv8 closely following (∼0.84), while YOLOv5/7 trail slightly (0.75–0.82).

(iii) Leaf is the most challenging class: On the leaf class, YOLOv8/11 are validated around 0.74–0.78, outperforming YOLOv7 (0.59) and staying YOLOv5 (∼0.76). This result is consistent with the aggregate metrics in Table 1, where anchor-free heads deliver stronger boundary quality on amorphous structures.

(iv) Generalization gaps and model behavior: YOLOv7 shows the largest train→val drop on Leaf class (0.66→0.59) as well as for other classes, a pattern that points to weaker generalization when faced with high-variability foliage. By contrast, YOLOv8/11 have almost no gaps (0,1) for all classes which is a sign of more stable optimization and a stronger inductive bias for thin or irregular boundaries. YOLOv5 occupies few gaps (≤0.01–0.02) but has comparatively strong results on all classes.

Overall, the class-level analysis reinforces our main conclusion: Anchor-free YOLO generations (v8/11) provide the best balance of accuracy and generalization, especially for classes with subtle edges and texture variability (the green and necrosis classes).

### 2.4. Comparative Prediction Performance and Key Insights

**Figure 5:**
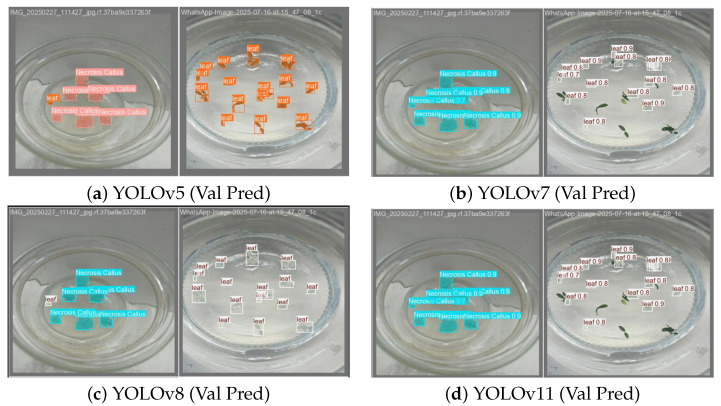
Qualitative segmentations across models (validation): (**a**) YOLOv5; (**b**) YOLOv7; (**c**) YOLOv8; and (**d**) YOLOv11. Anchor-free variants (YOLOv8/11) better preserve thin boundaries and reduce under/over-segmentation near petioles.

The validation outputs of four successive YOLO models show both the fidelity of boundaries and the main failure cases. Anchor-free models are able to retain thin or irregular structures and limit leakage into leaf veins. Anchor-based models, however, often fail to capture small emerging callus or mistakenly merge them with leaf regions under low-contrast conditions. The examples in Figure 5 demonstrate the practical strengths of anchor-free designs for complex plant tissue morphology and confirm their suitability for real-time culture monitoring.

The analysis yields three principal insights, foremost among them that anchor-free architectures (YOLOv8/11) surpass anchor-based models (YOLOv5/7) by delivering higher segmentation accuracy together with greater computational efficiency in callus tissue applications. Among the evaluated models, YOLOv8 demonstrates the highest accuracy and fastest inference, making it the most practical choice for real-time applications. In addition, persistent issues such as class imbalance and morphological variability, most evident in leaf tissues, continue to limit performance and point to the need for broader and more diverse training datasets.

## 3. Discussion

The comparative evaluation of YOLOv5/7/8/11 shows how successive architectural innovations influence segmentation accuracy, efficiency, and applicability in plant tissue culture. YOLOv5/7, as anchor-based variants, were historically important but proved less effective on irregular and amorphous callus tissues, since their reliance on predefined anchors limited boundary Precision and Recall. Among them, YOLOv7 had a heavy architecture that required considerably more computational resources but did not deliver proportional gains in accuracy.

In contrast, YOLOv8/11, which represents the anchor-free family, consistently outperformed earlier generations. YOLOv8 reached the best segmentation boundaries and efficiency since it included C2f modules and refined decoupled heads. The anchor-free paradigm, which removes the dependence on anchor priors, was particularly advantageous for amorphous and heterogeneous tissues that had highly irregular boundaries. Direct center and boundary regression provided stronger generalization to biological variability and reduced misclassification of overlapping or diffuse regions. In short, anchor-free models do not deal with the complexity of predefined anchors, which results in smoother optimization and fewer gradient updates. Their decouple heads learn through object centers and precise boundaries, leading to better localization. That is why anchor-free models work better on irregular callus shapes.

Per-class analysis showed that green callus was segmented most reliably, which was likely due to its strong contrast with surrounding tissue. Necrosis callus had moderate accuracy, while leaf structures remained the most difficult, which reflected both morphological variability and class imbalance. There are several factors that may cause difficulty in instance segmentation of leaf structure. Firstly, the leaf tissue has a smooth, wrinkled, and fragmented structure, which creates a complex morphological structure. Secondly, the leaf edges are often a slow sign of partial browning, which is very hard to distinguish from necrosis callus. Lastly, in dense cultures, leaf explants are partially overlaid on callus and necrosis tissues, which makes the estimation of boundary delineation very difficult. These findings confirmed the practical value of anchor-free designs for real-time monitoring in tissue culture, where segmentation precision and processing speed were both essential.

## 4. Materials and Methods

### 4.1. Successive Generations of YOLO Architectures

**Figure 6:**
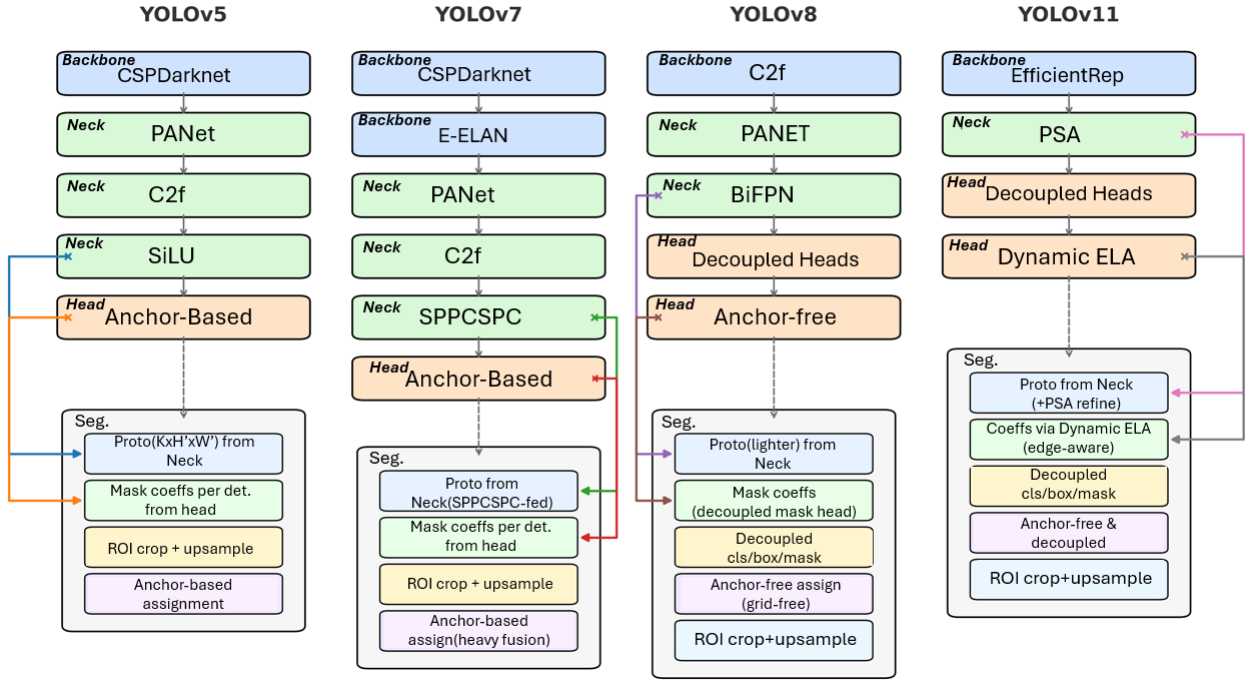
Architecture comparison of YOLO variants for instance segmentation. Each column shows Backbone → Neck → Head and the Seg. path. YOLOv5/7 rely on anchor-based heads, while YOLOv8/11 adopt anchor-free and decoupled designs.

YOLO frameworks contain three parts: the backbone, which extracts feature maps; the neck, which fuses multi-scale information; and the head, which outputs predictions such as classes, boxes, and masks. Across versions, the design has shifted from anchor-based models (YOLOv5/7) to leaner anchor-free and decoupled systems (YOLOv8/11) that improve performance in instance segmentation, as illustrated in Figure 6.

YOLOv5: A widely adopted real-time baseline using a Cross-Stage Partial Darknet (CSPDarknet) backbone, a Path Aggregation Network (PANet)-style neck, and Sigmoid Linear Unit (SiLU) activations. The segmentation branch relies on predefined anchors, where prototypes are produced by the neck and instance coefficients by the head, combined via anchor-based assignment before Region of Interest (ROI) cropping and upsampling [26].

YOLOv7: YOLOv7 introduced the Extended Efficient Layer Aggregation Network (E-ELAN), re-parameterized convolutions, and Spatial Pyramid Pooling with Cross-Stage Partial connections (SPPCSPC) for stronger multi-scale fusion. Although accuracy improves, it remains anchor-based and computationally heavy [27].

YOLOv8: YOLOv8 marked a paradigm shift to anchor-free prediction with decoupled heads for classification, box, and mask coefficients. Cross-Stage Partial with two convolutions and feature reuse (C2f) modules and a Bi-directional Feature Pyramid Network (BiFPN) neck improved feature aggregation. This design simplified label assignment and improved boundary fidelity for amorphous objects [28].

YOLOv11: The most recent generation integrates an Efficient Reparameterizable (EfficientRep) backbone and Parallel Spatial Attention (PSA) in the neck that refines prototype masks. A decoupled head with Dynamic Edge-aware Localization Adjustment (ELA) predicts coefficients. The system maintains an anchor-free design but achieves higher throughput with strong segmentation accuracy [29,30].

Overall, all YOLO generations share the same segmentation principle: prototype masks (*P*) from the neck are linearly combined with per-instance coefficients (α) from the head, cropped to the ROI, and upsampled to full resolution:

(1) $$M=\mathrm{Upsample}\;\left({\mathrm{Crop}}_{\mathrm{ROI}}\;\left(\sum_{i=1}^{k}\alpha_{i}P_{i}\right)\right)$$

The main differences lie in architectural choices (backbone, neck, and head) and whether detection is anchor-based (YOLOv5/7) or anchor-free (YOLOv8/11).

### 4.2. Plant Material

Three different lentil genotypes (Fırat-87, cagıl, and Tigris) were used as one data set in this study as the source material. Surface sterilization of lentil seeds was performed using 15% commercial bleach solution (equivalent to approximately 5% NaOCl) for 10 min. To eliminate the residual effects of bleach, the seeds were rinsed three times with sterile distilled water, each rinse lasting 5 min. The sterilized seeds were then cultured on Murashige and Skoog (MS) nutrient medium supplemented with 3% sucrose and solidified with 0.6% agar. Cultures were maintained for four weeks under controlled growth conditions to obtain healthy seedlings.

### 4.3. Callus Induction

**Figure 7:**
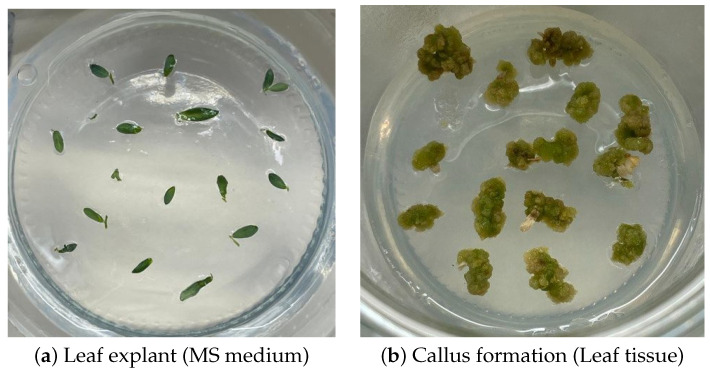
Representative images showing (**a**) a leaf explant after transfer to callus induction medium and (**b**) subsequent callus development under hormonal treatment.

Leaf explants excised from the regenerated seedlings were transferred to MS medium containing different combinations of the growth regulators 6-benzylaminopurine (BAP) at 0.5, 1.0, and 1.5 mg/L, and naphthaleneacetic acid (NAA) at 0.25, 0.50, and 1.0 mg/L (including a control group). Similar plant growths’ regulatory combination have been widely reported for efficient callus induction and regeneration in lentil and related legumes [31,32,33]. Machine learning approaches have also been proposed to predict callus induction efficiency in cereals, highlighting the importance of reproducible induction systems [34]. All cultures in our study were established in three biological replicates and maintained under standard growth conditions. Throughout the culture period, callus tissues that are developed on the explants were later photographed for dataset construction using a high-resolution digital camera positioned at a fixed distance of 17 cm inside a biosafety cabinet under uniform laboratory lighting, as seen Figure 7.

### 4.4. Dataset Preparation and Augmentation

**Figure 8:**
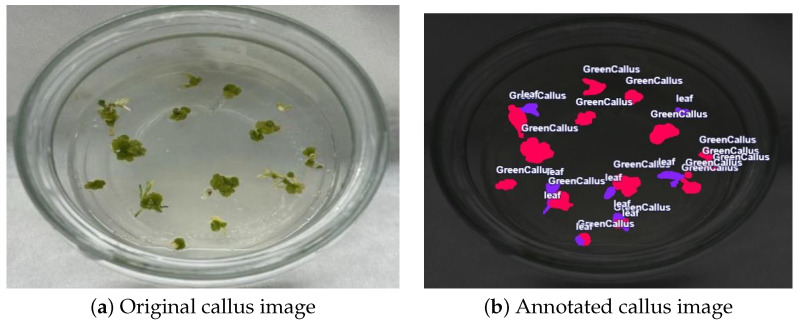
Example of manually annotated callus images using polygon masks. (**a**) Original image of a callus. (**b**) The corresponding annotated version, where a mask highlights the Region of Interest (ROI) for segmentation.

Lentil seeds were cultured for four weeks to obtain leaf explants. Callus formation (as described in Section 4.3) was then induced over an additional six-week leaf culture period. From all cultures, a total of 122 high-resolution images were obtained, and each image was manually annotated using polygon masks as illustrated in Figure 8. Three biologically relevant classes were defined: green callus with 478 instances (40.3%), necrosis callus with 425 instances (35.9%), and leaf tissue with 282 instances (23.8%), which yield a total of 1185 annotations.

**Figure 9:**
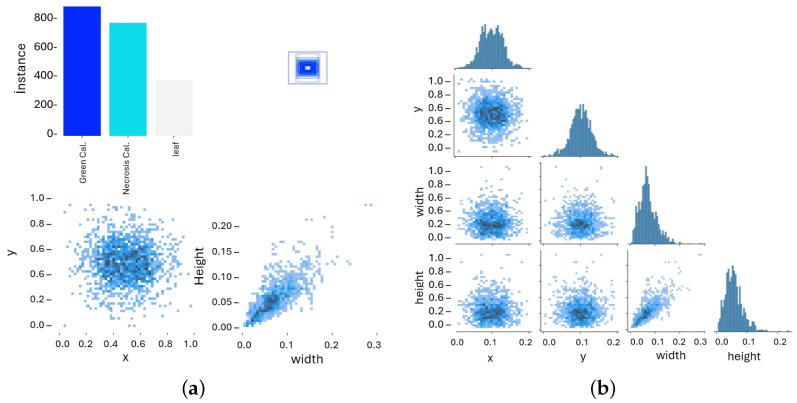
Dataset visualization: (**a**) example annotated image showing the three classes: green callus, necrosis callus, and leaf tissue; (**b**) class co-occurrence correlogram across the dataset, illustrating the distribution and co-presence of annotated categories.

This distribution reflects a slight imbalance, with green callus being the most represented, but it still provides a sufficient basis for model training and evaluation, as shown in Figure 9a,b.

**Figure 10:**
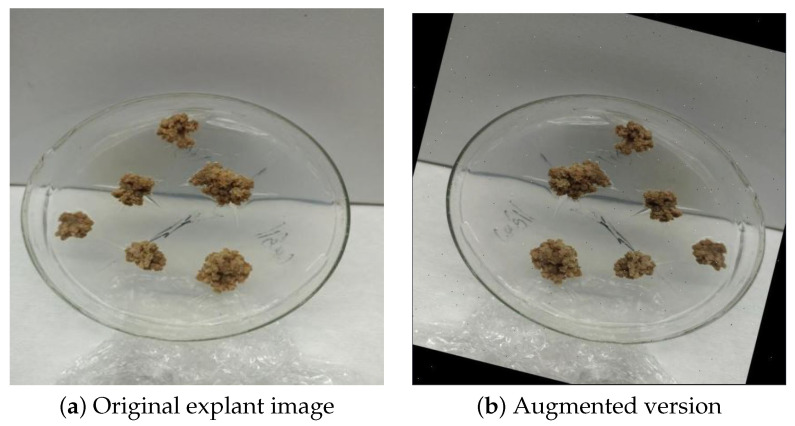
Example of a preprocessing and augmentation pipeline. (**a**) Original image and (**b**) the corresponding augmented version with auto-orient, resize (640 × 640), horizontal flip, $-14^{\circ }$ rotation, −18% saturation, −4% exposure, and 0.05% noise.

To enhance model robustness and generalization, data augmentation techniques were applied. All images were resized to 640×640 pixels prior to training, and up to three augmentations were applied per training example. These included horizontal and vertical flips, random rotations between $-15^{\circ }$ and $+15^{\circ }$, saturation adjustments in the range of ±25%, exposure (brightness) perturbations within ±10%, and additive noise affecting up to 0.1% of image pixels. An example is illustrated in Figure 10.

**Figure 11:**
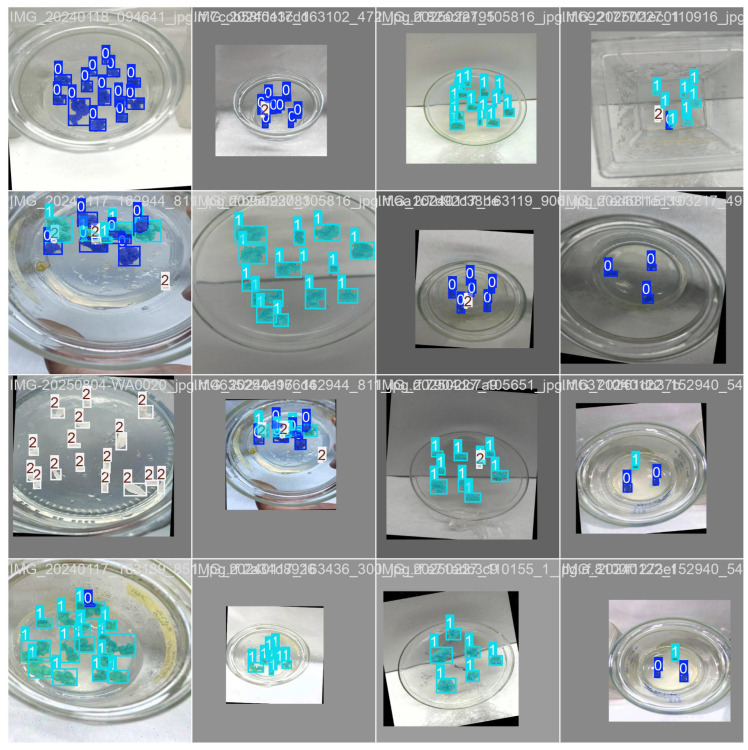
Training batch that shows the three classes: green callus (0), necrosis callus (1), and leaf tissue (2).

Such augmentations expanded the appearance space while preserving the biological meaning of annotations. Mosaic augmentation was additionally employed for YOLOv5 and YOLOv7 but disabled in the final 10 epochs for YOLOv8/11, as anchor-free models benefit from more stable late-stage optimization. Following augmentation, the dataset size increased to 254 images with augmented variants. A stratified split was applied, resulting in 75% training, 15% validation, and 10% testing. It is important to note that augmentation did not introduce new biological specimens. The total number of unique annotations remained 1185; however, after augmentation, these masks were presented to the model in transformed form, effectively doubling the number of training instances seen during optimization. An example of the obtained data is demonstrated in Figure 11.

### 4.5. Evaluation Metrics

In this work, model performance across classes was assessed using common segmentation metrics, including mean Average Precision (mAP), Dice coefficient, Precision, Recall, and Intersection over Union (IoU). The IoU for class *i* is defined as

(2) $$IoU_{i}=\frac{TP_{i}}{TP_{i}+FP_{i}+FN_{i}}$$

where $TP_{i}$, $FP_{i}$, and $FN_{i}$ are true positives, false positives, and false negatives for class *i*. This formulation follows the Jaccard index [35].

The Dice coefficient (equivalent to the F1 score at the pixel level) is

(3) $$Dice=\frac{2TP}{2TP+FP+FN}$$

which originates from Sørensen’s similarity index [36].

Precision and Recall are computed as

(4) $$Precision=\frac{TP}{TP+FP},\;\;\;\;Recall=\frac{TP}{TP+FN}$$

Finally, the mean Average Precision (mAP) is reported at multiple IoU thresholds, ranging from 0.5 to 0.95 with increments of 0.05:

(5) $$mAP=\frac{1}{\left|T\right|}\sum_{t\in T}AP_{t}$$

where $AP_{t}$ is the Average Precision at threshold *t* and *T* is the set of thresholds. This formulation follows the PASCAL VOC evaluation protocol [37].

These metrics provide different aspects of model behavior. IoU and Dice focus on the fidelity of spatial overlap between predictions and ground truth, whereas Precision and Recall reflect the reliability of classification decisions. Meanwhile, mAP integrates performance across multiple thresholds to give a balanced view of detection and segmentation accuracy. As noted in the recent literature, meaningful evaluation also requires weighing accuracy against computational efficiency [38].

### 4.6. Computational Setup

#### 4.6.1. Computational Environment

The experiments were carried out on a cloud-based system equipped with an NVIDIA Tesla T4 GPU (16 GB VRAM), 25 GB of RAM, and dual CPUs. The utilized software for training and validation was Ubuntu 20.04 with Python 3.10. YOLOv5/7 were implemented using PyTorch 1.13.1, while YOLOv8/11 were trained with the Ultralytics framework (version 8.3.40). Dataset preparation and annotation exports were managed through Roboflow.

#### 4.6.2. Training Configuration

To allow for consistent comparisons, all models were trained under identical hyperparameter settings. Each architecture was optimized for 100 epochs using a batch size of 16 and an input resolution of 640×640 pixels. The AdamW optimizer was applied with an initial learning rate of $1\times 10^{-3}$. Training made use of label caching and mixed-precision computation (FP16) to improve throughput. Standard augmentation strategies included horizontal and vertical flips, adjustments to brightness and contrast, Gaussian and median blurring, optional grayscale conversion, and CLAHE. For the anchor-free variants (YOLOv8/11), mosaic augmentation was turned off during the last ten epochs to promote more stable convergence in later training stages.

#### 4.6.3. Model-Specific Setup

Each YOLO generation required distinct modifications to align with the characteristics of its architecture. YOLOv5 was trained through its official segmentation branch, which was initialized with COCO-pretrained weights. YOLOv7 was implemented with its extended segmentation framework, and this implementation necessitated minor modifications to the plotting utilities in order to ensure compatibility with updated libraries. YOLOv8 adopted the anchor-free decoupled head, and its training pipeline was streamlined through the Ultralytics interface, which provided integrated monitoring of both loss curves and validation performance. YOLOv11 followed a training routine comparable to earlier versions; however, it incorporated the EfficientRep backbone together with Parallel Spatial Attention, which enabled the model to converge more rapidly than its predecessors. The architecture of YOLOv7 was more complex, and as a result, it took longer to train. YOLOv8/11, on the other hand, converged faster and stabilized validation accuracy in fewer epochs.

#### 4.6.4. Statistical Significance

To check if the differences in accuracy and efficiency among the models were meaningful, we relied on relative improvement measures. The gain in accuracy of model *A* compared with model *B* was defined as

(6) $$\Delta mAP=\frac{mAP_{A}-mAP_{B}}{mAP_{B}}\times 100\%$$

where $mAP_{A}$ and $mAP_{B}$ denote the mean Average Precision values of the two models under comparison.

The relative improvement in inference efficiency was defined as

(7) $$\Delta FPS=\frac{FPS_{A}-FPS_{B}}{FPS_{B}}\times 100\%$$

where $FPS_{A}$ and $FPS_{B}$ represent the frame rates of the respective models.

These formulations serve as a consistent framework for measuring improvements in both accuracy and inference speed. In our experiments, the differences appeared across multiple validation splits, which shows that the observed advantages are not the result of random variation but instead stem from the architectures themselves.

## 5. Conclusions

This work delivered the first systematic study of successive YOLO architectures applied to instance segmentation of lentil callus tissues in plant tissue culture. The evaluation was carried out on 122 high-resolution images with 1185 annotations spanning three biologically relevant classes. Across this dataset, anchor-free models (YOLOv8/11) performed more effectively than anchor-based alternatives (YOLOv5/7) in both segmentation accuracy and computational efficiency. YOLOv8 offered the most precise boundary delineation and balanced high accuracy with faster inference, making it the more practical option for real-time analysis. Overall, the findings show that anchor-free designs provide a stronger fit for the irregular and amorphous morphology that characterizes callus tissues. More broadly, the results show that deep learning–based instance segmentation can improve reproducibility and scalability in plant tissue culture monitoring.

## Figures and Tables

**Table 1:** Segmentation performance (mask mAP, Precision, Recall, Dice), efficiency metrics, and training time on an NVIDIA Tesla T4 GPU.

Model	mAP50	mAP50–95	Mask P	Mask R	Dice	Params (M)	FLOPs (G)	Train Time	FPS
YOLOv5	0.831	0.573	0.863	0.750	0.803	7.4	25.7	0.214 h	35
YOLOv7	0.772	0.515	0.797	0.682	0.734	37.9	141.9	0.652 h	28
YOLOv8	0.855	0.599	0.877	0.781	0.826	11.8	39.9	0.214 h	166
YOLOv11	0.832	0.581	0.851	0.782	0.816	10.1	32.8	0.214 h	133

## Data Availability

The dataset used in this study, including annotated images for callus detection, is publicly available and can be accessed at Roboflow Universe: https://universe.roboflow.com/yunus-7v2b5/callus-hug7d, accessed on 13 September 2025. This repository contains all images and annotations generated and analyzed during the current study. No additional proprietary or restricted datasets were used.

## Abbreviations

The following abbreviations are used in this manuscript:

AI	Artificial Intelligence
BAP	6-benzylaminopurine
BiFPN	Bi-directional Feature Pyramid Network
C2f	Cross-Stage Partial with two convolutions and feature reuse
CNN	Convolutional Neural Network
CSPDarknet	Cross-Stage Partial Darknet
ELA	Edge-aware Localization Adjustment
E-ELAN	Extended Efficient Layer Aggregation Network
FPS	Frames Per Second
FLOPs	Floating Point Operations
IoU	Intersection over Union
mAP	mean Average Precision
MS medium	Murashige and Skoog nutrient medium
NAA	Naphthaleneacetic Acid
PANet	Path Aggregation Network
PSA	Parallel Spatial Attention
ROI	Region of Interest
SiLU	Sigmoid Linear Unit
SPPCSPC	Spatial Pyramid Pooling with Cross-Stage Partial connections
YOLO	You Only Look Once

## References

[1] Thorpe T.A. (2007). History of plant tissue culture. Mol. Biotechnol..

[2] Pasternak T.P., Steinmacher D. (2024). Plant Growth Regulation in Cell and Tissue Culture In Vitro. Plants.

[3] Nagata T., Takebe I. (1971). Plating of isolated tobacco mesophyll protoplasts on agar medium. Planta.

[4] Fehér A. (2019). Callus, dedifferentiation, totipotency, somatic embryogenesis. Front. Plant Sci..

[5] FAO (2019). The International Year of Pulses: Final Report.

[6] Bagheri A., Ghasemi Omraan V.O., Hatefi S. (2012). Indirect in vitro regeneration of lentil. J. Plant Mol. Breeding.

[7] Lin T.-Y., Maire M., Belongie S., Hays J., Perona P., Ramanan D., Dollár P., Zitnick C.L. Microsoft COCO: Common Objects in Context. Proceedings of the Computer Vision—ECCV 2014.

[8] Kamilaris A., Prenafeta-Boldú F.X. (2018). Deep learning in agriculture: A survey. Comput. Electron. Agric..

[9] Bochkovskiy A., Wang C.-Y., Liao H.-Y.M. (2020). YOLOv4: Optimal speed and accuracy of object detection. arXiv.

[10] Alif M., Hussain S. (2024). YOLO object detection algorithms from v1 to v10: A review with applications. Artif. Intell. Agric..

[11] Zou Z., Chen K., Shi Z., Guo Y., Ye J. (2023). Object Detection in 20 Years: A Survey. Proc. IEEE.

[12] Guo C., Tan F. (2025). SWRD-YOLO: A Lightweight Instance Segmentation Model for Estimating Rice Lodging Degree in UAV Remote Sensing Images with Real-Time Edge Deployment. Agriculture.

[13] Zhao L., Olivier K., Chen L. (2025). An Automated Image Segmentation, Annotation, and Detection Framework Integrating SAM and YOLOv8. Agronomy.

[14] Zhang D., Lu R., Guo Z., Yang Z., Wang S., Hu X. (2024). F-YOLOv8n-seg: A Lightweight Model for Weed Meristem Localization. Agronomy.

[15] Sonawane D., Patil S. (2025). Crop–Weed Segmentation Using YOLOv8 for Smart Farming. Smart Agric. Syst..

[16] Sapkota R., Karkee M. (2024). Comparing YOLOv11 and YOLOv8 for Instance Segmentation of Immature Green Fruitlets in Orchard Environments. arXiv.

[17] Ma H., Zhao Q., Zhang R., Hao C., Dong W., Zhang X., Li F., Xue X., Sun G. (2025). YOLOv11-GSF: An optimized deep learning model for strawberry ripeness detection in agriculture. Front. Plant Sci..

[18] Khan A.T., Jensen S.M. (2025). Leaf Segmentation across Multiple Crops Using YOLOv11. Agriculture.

[19] Zhu H., Wang D., Wei Y., Wang P., Su M. (2025). YOLOv8-CMS: A High-Accuracy Deep Learning Model for Automated Citrus Leaf Disease Classification and Grading. Plant Methods.

[20] Daraghmi Y.A., Naser W., Daraghmi E.Y., Fouchal H. (2025). Drone-Assisted Plant Stress Detection Using Deep Learning. Agronomy.

[21] Tian Z., Shen C., Chen H., He T. FCOS: Fully Convolutional One-Stage Object Detection. Proceedings of the IEEE/CVF International Conference on Computer Vision (ICCV).

[22] Zhou X., Wang D., Krähenbühl P. Objects as Points. Proceedings of the IEEE/CVF Conference on Computer Vision and Pattern Recognition (CVPR).

[23] Carion N., Massa F., Synnaeve G., Usunier N., Kirillov A., Zagoruyko S. End-to-End Object Detection with Transformers. Proceedings of the Computer Vision—ECCV 2020.

[24] Caicedo J.C., Goodman A., Karhohs K.W., Cimini B.A., Ackerman J., Haghighi M., Heng C., Becker T., Doan M., McQuin C. (2019). Nucleus segmentation across imaging experiments: The 2018 Data Science Bowl. Nat. Methods.

[25] Moen E., Bannon D., Kudo T., Graf W., Covert M., Valen D.V. (2019). Deep learning for cellular image analysis. Nat. Methods.

[26] Jocher G., Chaurasia A., Qiu J. (2020). YOLOv5: Implementation of You Only Look Once in PyTorch. GitHub Repository. [Online]. https://github.com/ultralytics/yolov5.

[27] Wang C.-Y., Bochkovskiy A., Liao H.-Y.M. (2022). YOLOv7: Trainable bag-of-freebies sets new state-of-the-art for real-time object detectors. arXiv.

[28] Ultralytics (2023). YOLOv8: Next-Generation YOLO Models. Ultralytics Documentation. [Online]. https://docs.ultralytics.com.

[29] Khanam R., Hussain M. (2024). YOLOv11: An Overview of the Key Architectural Enhancements. arXiv.

[30] He W., Zhou Y., Liu L., Ma J. (2024). YOLOv11-Seg: Instance Segmentation for Construction Site Monitoring. Buildings.

[31] Polanco C., Ruiz M.L. (2002). Efficient in vitro callus induction and plant regeneration of lentil. Plant Cell Rep..

[32] Barik D.P., Naik P., Mohapatra P.K. (2005). In vitro callus induction and plant regeneration in *Lens culinaris* Medik. Biol. Plant..

[33] Rai R., Shekhawat A., Singh V. (2011). Genotypic variability in callus induction and plant regeneration in lentil (*Lens culinaris* Medik.). Legume Res..

[34] Siddique A.B., Islam S.M.A. (2015). Predicting rice callus induction using machine learning models. Plant Tissue Cult. Biotechnol..

[35] Jaccard P. (1901). Étude comparative de la distribution florale dans une portion des Alpes et des Jura. Bull. Soc. Vaudoise Sci. Nat..

[36] Sørensen T. (1948). A method of establishing groups of equal amplitude in plant sociology based on similarity of species content. Biol. Skr..

[37] Everingham M., Gool L.V., Williams C.K.I., Winn J., Zisserman A. (2010). The PASCAL Visual Object Classes (VOC) Challenge. Int. J. Comput. Vis..

[38] Padilla R., Netto S.L., da Silva E.A.B. A Survey on Performance Metrics for Object-Detection Algorithms. Proceedings of the 2020 International Conference on Systems, Signals and Image Processing (IWSSIP).

